# Single Cell Analysis of Reversibility of the Cell Death Program in Ethanol-Treated Neuronal PC12 Cells

**DOI:** 10.3390/ijms23052650

**Published:** 2022-02-28

**Authors:** Wenting You, Tos T. J. M. Berendschot, Kèvin Knoops, Marc A. M. J. van Zandvoort, Carroll A. B. Webers, Chris P. M. Reutelingsperger, Theo G. M. F. Gorgels

**Affiliations:** 1 University Eye Clinic Maastricht UMC+, Maastricht University Medical Center+, 6229 HX Maastricht, The Netherlands; w.you@maastrichtuniversity.nl (W.Y.); t.berendschot@mumc.nl (T.T.J.M.B.); c.webers@mumc.nl (C.A.B.W.); 2 Department of Biochemistry, CARIM School for Cardiovascular Disease, Maastricht University, 6229 ER Maastricht, The Netherlands; 3 Department of Mental Health and Neuroscience, Maastricht University, 6229 ER Maastricht, The Netherlands; 4 The Maastricht Multimodal Molecular Imaging Institute, Maastricht University, 6229 ER Maastricht, The Netherlands; k.knoops@maastrichtuniversity.nl; 5 Department of Molecular Cell Biology, CARIM School for Cardiovascular Disease, Maastricht University, 6229 ER Maastricht, The Netherlands; mamj.vanzandvoort@maastrichtuniversity.nl; 6 Institute of Molecular Cardiovascular Research, Universitätsklinikum Aachen, 52074 Aachen, Germany

**Keywords:** cell death program, neurite retraction, neurite regeneration, nuclear shrinkage, mitochondrial fragmentation, PS exposure, PC12 cells

## Abstract

Neurodegenerative diseases are generally characterized clinically by the selective loss of a distinct subset of neurons and a slow progressive course. Mounting evidence in vivo indicates that large numbers of neurons pass through a long period of injury and dysfunction before the actual death of the cells. Whether these dying neurons can be rescued and return to a normal, functional state is uncertain. In the present study, we explored the reversibility of the neuronal cell death pathway at various stages by monitoring the dynamics of single cells with high-resolution live-cell spinning disk confocal microscopy in an in vitro neuronal cell death model. We exposed differentiated neuronal PC12 cells to ethanol as our cell death model. Results showed that exposure to 5% ethanol for 24 h induced cell death in >70% of the cells. Ethanol treatment for 3 h already induced cellular changes and damage such as reactive oxygen species generation, elevation of intracellular Ca^2+^ level, phosphatidylserine exposure, nuclear shrinkage, DNA damage, mitochondrial fragmentation and membrane potential loss, and retraction of neurites. These phenomena are often associated with programmed cell death. Importantly, after removing ethanol and further culturing these damaged cells in fresh culture medium, cells recovered from all these cell injuries and generated new neurites. Moreover, results indicated that this recovery was not dependent on exogenous NGF and other growth factors in the cell culture medium. Overall, our results suggest that targeting dying neurons can be an effective therapeutic strategy in neurodegenerative diseases.

## 1. Introduction

Neurodegenerative diseases have a big impact on patients, their families and on society as a whole. Clinically, these diseases are generally characterized by the selective loss of a distinct subset of neurons and their slow, progressive course [1,2,3]. Since neurons are post-mitotic, the capacity for replacing lost cells is very limited. Current efforts on combatting neurodegenerative diseases are aimed at protecting the neurons and preventing their death. Mounting evidence in vivo has indicated that large numbers of neurons pass through a long period in which they show pathological changes before ultimately the death of the cells occurs [4,5,6,7,8]. Interestingly, some in vitro studies revealed that a variety of cell types can recover from the injury and imminent cell death process, such as primary mouse liver cells and macrophages, primary rat cardiomyocytes, NIH3T3 fibroblasts, and various tumor cells (Hela, HepG2, A375, MCF7 cells) [9,10,11,12]. Whether this is also possible for neurons and whether dying neurons can be rescued and return to a functional state is still uncertain. This may signify the potential to prevent progression of the disease or even the regeneration and restoration of function.

Programmed cell death (PCD) is a crucial physiological process during early development of the nervous system, since elimination of excess neuronal cells through PCD is essential for the establishment and maintenance of the functional neural network [13,14]. However, once the nervous system is fully established, PCD pathways become highly restricted in mature neurons, as these valuable cells need to perform their often unique, functions over a lifetime [15]. Studies on diverse tissues and cells have revealed that mature neurons have adopted multiple strategies to maintain cellular homeostasis and avoid aberrant cell death [16,17]. These intrinsic anti-apoptotic mechanisms might enable neurons to withstand a variety of insults and even rescue neuronal cells from injury. Cell death may be the final solution for a neuron only when the intrinsic survival factors are overwhelmed, either acutely or chronically. To explore the possibility of rescuing injured and dying neurons, a detailed analysis of the neuronal cell death pathway is needed, specifically exploring the potential of reversibility of cell death along the different phases of the cell death program.

There are many common mechanisms and features shared by pathologically distinct neurodegenerative diseases, such as reactive oxidative stress, dysregulation of intracellular calcium, excitotoxicity, mitochondrial dysfunction, DNA damage, inflammation, transcription and translation disruption, disruption of the protein quality control system, and synaptic dysfunction [18,19]. Changes like these often precede final neuronal cell loss. To study whether there is a time window for rescuing dying neuronal cells, we monitored the dynamics of single neuronal PC12 cells with high-resolution live-cell spinning disk confocal microscopy, which enabled us to track and record the response of individual cells before, during, and after exposure to a cell death stimulus of ethanol. Surprisingly, we found that after removal of the ethanol, more than 90% of the cells recovered from ethanol induced reactive oxygen species (ROS) generation, persistent elevation of intracellular Ca^2+^ level, phosphatidylserine (PS) exposure, nuclear shrinkage, DNA damage, and structural and functional injury of the mitochondria. Also, we observed a fast growth of new neurites.

## 2. Results

### 2.1. Ethanol Induced Cell Death on Neuronal PC12 Cells

**Figure 1:**
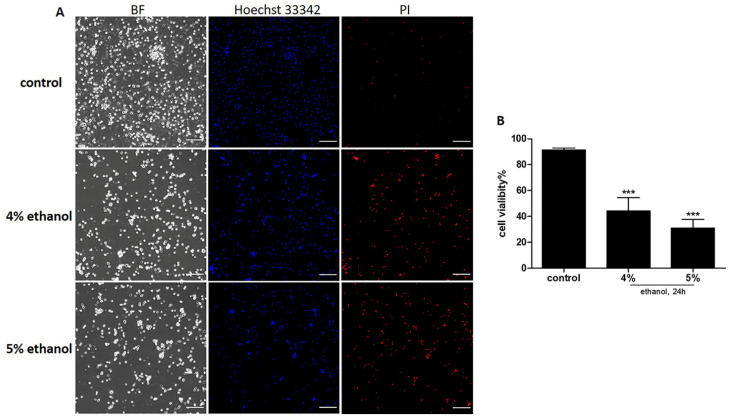
Effect of ethanol on neuronal PC12 cell viability. (**A**) Cells were exposed to 4% or 5% ethanol (vol/vol) for 24 h. Cell viability was assessed by Hoechst 33342/PI double staining. (**B**) Quantification of cell viability. Data are presented as the mean ± SD (*n* = 3). *** *p* < 0.001, compared with the control group. Scale bar: 200 µm.

In order to explore the capacity of NGF-differentiated PC12 cells to reverse activated PCD pathways we first exposed differentiated PC12 cells to ethanol and recorded cell viability. It has been reported that ethanol treatment induces widespread apoptotic neurodegeneration both in vivo and in vitro [20,21]. 24 h of treatment with 4% and 5% ethanol reduced the number of viable cells, with 55% and 70%, respectively, as measured by PI-uptake (Figure 1). In this cell death program model, we investigated which events and phenotypic hallmarks occur successively during the execution of the cell death program. To study the reversibility of this cell death program and the capacity of neuronal PC12 cells to recover, we removed the toxic stimulus by changing the medium to fresh medium without ethanol.

### 2.2. Neurites Retraction and Regeneration Analysis

#### 2.2.1. Reversible Loss of Neurites Induced by Ethanol in PC12 Cells

**Figure 2:**
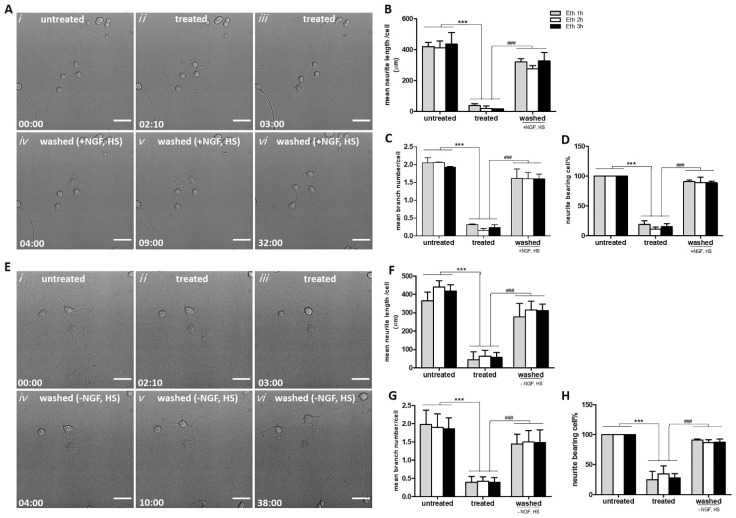
Neurite regeneration in medium with or without NGF and HS after removing ethanol. (**A**) Real-time imaging of the same cells, before 5% ethanol (vol/vol) exposure (untreated, *i*), treated for 1 h (treated, *ii* and *iii*), subsequently washed and further incubated with fresh differentiating medium for another 29 h (washed, *iv*–*vi*). Scale bar: 50 µm. A corresponding movie is available as Supplemental Movie 1. (**B**–**D**) results of quantification of mean neurite length, mean branch number per cell, and percentage of neurite bearing cells. (**E**) Real-time imaging of the same cells, before 5% ethanol (vol/vol) exposure (untreated, *i*), treated for 1 h (treated, *ii* and *iii*), subsequently washed and further incubated with fresh culture medium without NGF and HS for another 35 h (washed, *iv*–*vi*). Scale bar: 50 µm. A corresponding movie is available as Supplemental Movie 2. (**F**–**H**) results of quantification of mean neurite length, mean branch number per cell, and percentage of neurite bearing cells. *** *p* < 0.001, compared with untreated group; ^###^ *p* < 0.001, compared with treated group. All data are reported as mean ± SD (*n* = 3–5, at least 100 cells were quantified in each group). Time presented as hr:min. Eth: ethanol.

We observed that ethanol induced neurite retraction and degeneration within 1 h after the start of the exposure. Figure 2A shows that 5% ethanol caused significant neurite loss as indicated by reduced length (Figure 2B) and branching (Figure 2C) of about 80% of the PC12 cells (Figure 2D). Next, we investigated whether the response of neurite retraction was reversible. Therefore, we washed away the ethanol after 1, 2 and 3 h of exposure and cultured the cells for 24 h in ethanol-free medium. By tracking single cells, we found that 90% of the cells that had lost their neurites were able to develop new ones (Figure 2D) with comparable length (Figure 2B) and branching (Figure 2C) within 24 h (Supplemental Movie S1).

#### 2.2.2. Neurite Regrowth Does Not Depend on NGF and HS in the Culture Medium

Since undifferentiated PC12 cells depend on exogenously added NGF to grow neurites [22], we wondered whether neurite regeneration after the removing of ethanol was dependent on NGF or other growth factors present in the medium. The medium used for culturing the differentiated PC12 cells contained 50 ng/mL NGF, 1% HS and no FBS. To answer this question, we washed the cells after exposure with 5% ethanol and cultured them further in fresh medium without NGF and HS. We observed that neuronal PC12 cells, which had lost all neurites by ethanol treatment for 1, 2, or 3 h, could still develop new neurites and recover to normal morphology in 1d (Figure 2E–H, Supplemental Movie 2). This indicates that neurite regeneration of ethanol treated neuronal PC12 cells is not dependent on exogenously added NGF and other growth factors.

### 2.3. Elevation of Intracellular ROS and Ca^2+^ Induced by Ethanol in Neuronal PC12 Cells

**Figure 3:**
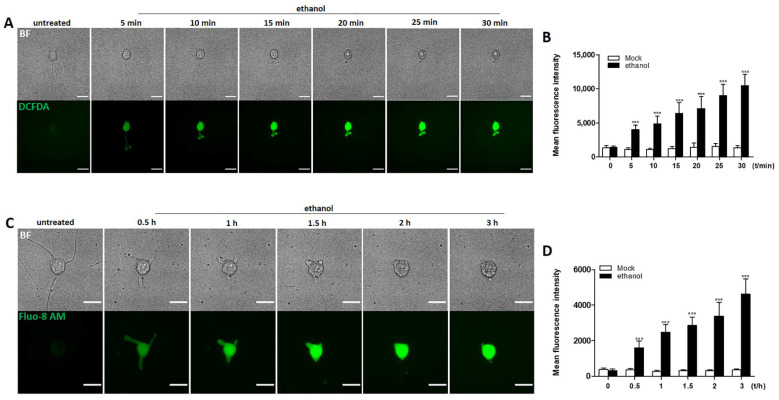
ROS generation and Ca^2+^ influx induced by ethanol in neuronal PC12 cells. (**A**) ROS generation indicated by DCFDA; cells were treated with 5% ethanol (vol/vol); images were acquired every 5 min. Scale bar: 50 µm. (**B**) quantification of mean fluorescence intensity based on DCFDA staining. (**C**) Ca^2+^ influx indicated by Fluo-8 AM; cells were treated 5% ethanol (5%, vol/vol); images were acquired every 30 min. Scale bar: 50 µm. (**D**) quantification of mean fluorescence intensity based on Fluo-8 AM. Mock treated cells were imaged in parallel to ensure that imaging and staining were not cytotoxic. All data are reported as mean ± SD (*n* = 3). *** *p* < 0.001, compared with untreated group.

PC12 cells and primary cortical neurons show increased oxidative stress when exposed to 100 mM ethanol for four days [23]. Oxidative stress can give rise in cytosolic Ca^2+^ levels [24]. We examined whether these responses occurred in neuronal PC12 cells during ethanol induced neurite retraction. We assessed baseline- and ethanol- induced levels of intracellular ROS and cytoplasmic Ca^2+^ levels using DCFDA and Fluo-8 AM, respectively. Figure 3 shows that ethanol gradually increased both the level of intracellular ROS (Figure 3A,B) and the level of cytoplasmic Ca^2+^ (Figure 3C,D) during the time course of 0.5 and 3 h, respectively. The increase in both markers accompanied the ethanol induced neurite retraction.

### 2.4. Recovery of Nuclear Shrinkage and DNA Damage with or without Exogenous NGF

**Figure 4:**
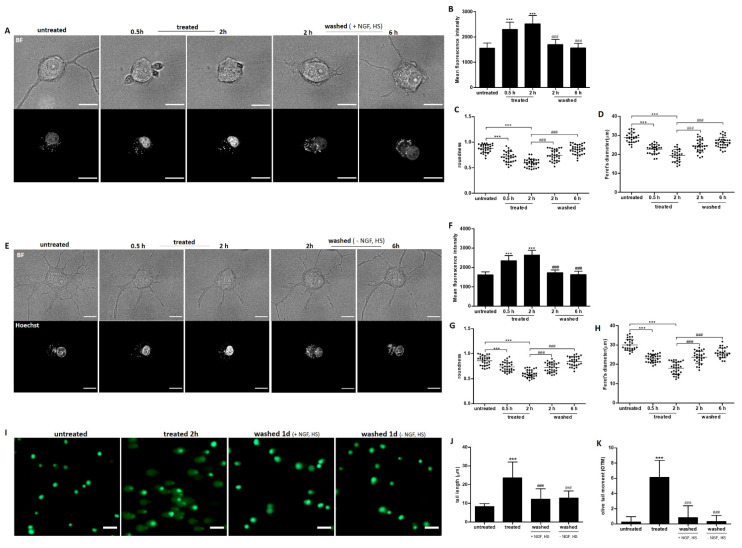
Recovery of nuclear shrinkage and DNA damage after culturing in medium with or without NGF and HS, after removing ethanol. (**A**,**E**) live cell imaging of the same neuronal PC12 cell before ethanol (5%, vol/vol) treatment (untreated), treated with ethanol for 0.5 and 2 h (treated), and after washing and incubation in fresh medium with or without NGF and HS for 2 and 6 h to allow recovery (washed). Nuclei were visualized by staining with Hoechst 33342. Scale bar: 50 µm. (**B**–**D**) and (**F**–**H**), results of quantification of mean fluorescence intensity of Hoechst, sphericity and the Feret’s diameter indicated nuclear condensation and morphology changes. (**I**) representative images of comet assay of neuronal PC12 cells before 5% ethanol treatment (untreated), treated for 2 h (treated), and treated cells which were washed and further incubated with fresh culture medium with or without NGF, HS for another 24 h (washed). Scale bar: 20 µm. (**J**,**K**), Statistical analysis of tail length (µm) and olive tail moment (OTM) with Comet Assay IV. Values represent the mean ± SD of individual cells in three independent experiments. *** p < 0.001, compared with untreated group; *^###^ p* < 0.001, compared with the group which was treated for 2 h.

Ethanol induced nuclear shrinkage and internucleosomal DNA fragmentation of rat cortical neurons [25]. By staining the nuclei of neuronal PC12 cells with Hoechst 33342 (Hoechst) and imaging single cells to track the shape and size of the nucleus, we observed neurite retraction and significant nuclear shrinkage and condensation after 30 min of exposure to ethanol (Figure 4A). Deformed nuclei recovered to normal morphology together with the development of new neurites after removal of ethanol (Figure 4A). Quantification of nuclear fluorescence intensity and size using Image J confirmed reversibility of the nuclear changes that induced by ethanol (Figure 4B–D). Importantly, the recovery of the nuclear changes was not dependent on exogenously added NGF and HS (Figure 4E–H).

To further investigate whether ethanol induced DNA damage, we used the comet assay, a sensitive, versatile and simple technique for the evaluation of DNA single and double strand breaks [26]. Ethanol caused significant DNA damage of neuronal PC12 cells after 2 h, as indicated by the increase of tail length and olive tail moment (Figure 4I–K). The DNA damage was repaired within one day after removal of the ethanol by washing (Figure 4I–K). This recovery was not dependent on exogenously added NGF and HS. These results indicate that neuronal cells have the capacity to repair DNA damage and appear to recover to normal after the cell death stimulus is removed.

### 2.5. Recovery of Mitochondrial Fragmentation and Membrane Potential Loss with or without Exogenous NGF

**Figure 5:**
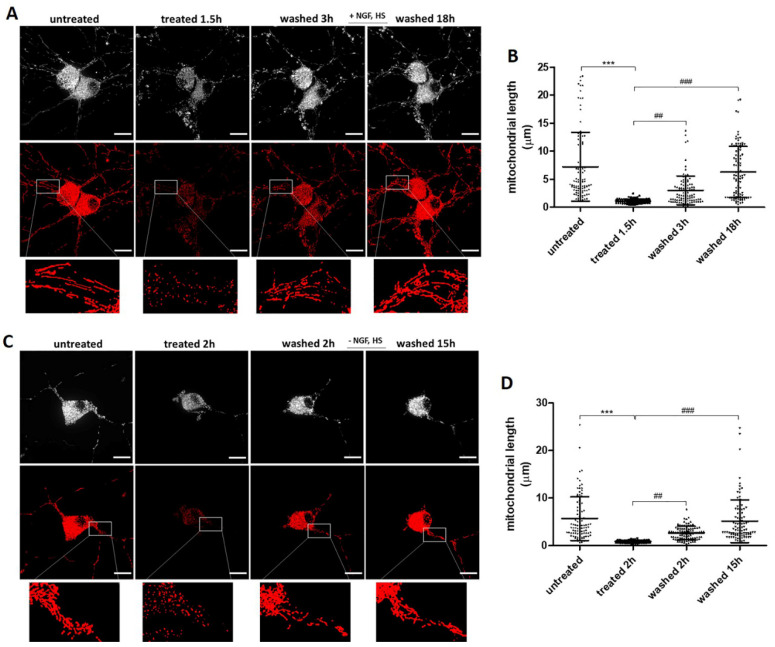
Reversibility of mitochondrial fragmentation after removing ethanol. (**A**,**C**) live cell imaging of the same neuronal PC12 cell, before ethanol (5%, vol/vol) treatment (untreated), treated with ethanol (treated), and after washing and incubation in fresh medium with or without NGF and HS to allow recovery (washed). Mitochondria were visualized by staining with TMRM. Grey: original fluorescence images; red: 3D projections of masked images. Scale bar: 50 µm. (**B**,**D**) results of quantification of mitochondrial length. Data are presented as the mean ± SD (*n* = 3). *** p < 0.001, compared with untreated group; *^##^ p* < 0.01, *^###^ p* *<* 0.001, compared with treated group.

Mitochondria are involved in ATP synthesis and cellular Ca^2+^ homeostasis, and are the primary source of intracellular ROS [27]. As we observed an increase in intracellular ROS and Ca^2+^ in ethanol treated neuronal PC12 cells, we next looked for mitochondrial changes by staining cells with Tetramethylrhodamine (TMRM). TMRM is a fluorescent lipophilic cation that accumulates within the mitochondrial matrix in a membrane potential-dependent manner where it produces a mitochondrial-specific fluorescent signal enabling semi-quantitative analysis of mitochondrial morphology and function [28]. Figure 5A shows that untreated neuronal PC12 cells have an extensively interconnected mitochondrial network. Exposure to 5% ethanol induced significant fragmentation of the mitochondrial network within 1.5–2 h. Consistently, the mean mitochondrial length decreased from about 7.24 µm to 1.33 µm in that time period (Figure 5B). Interestingly, the fragmented mitochondria recovered to the filamentous, elongated and interconnected structure after removal of ethanol and further culturing in medium (Figure 5A,B). Recovery of the mitochondria was not dependent on exogenously added NGF and HS (Figure 5C,D).

**Figure 6:**
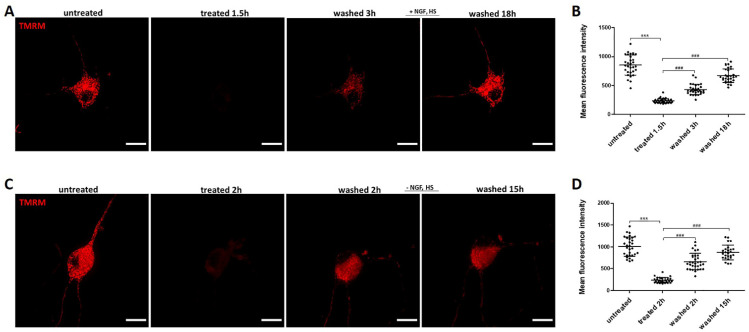
Recovery of mitochondrial membrane potential loss with or without exogenous NGF. (**A**,**C**) live cell imaging of the same neuronal PC12 cell, before ethanol (5%, vol/vol) treatment (untreated), treated with ethanol (treated), and after washing and incubation in fresh culture medium with or without NGF and HS to allow recovery (washed). Mitochondrial membrane potential was visualized by staining with TMRM. Scale bar: 50 µm. (**B**,**D**) TMRM fluorescence intensity was quantified with max intensity of 3D stacks in individual cells. Data are presented as the mean ± SD (n = 3). *** *p* < 0.001, compared with untreated group; *^###^ p* < 0.001, compared with the treated group.

Mitochondrial function is tightly linked to their morphology. We evaluated the mitochondrial membrane potential (Δψm) by quantifying the fluorescence signal intensity of TMRM stained mitochondria and observed that ethanol induced a significant Δψm reduction within 1.5–2 h, indicating loss of mitochondrial function (Figure 6A,B). As observed for morphology, mitochondrial function recovered when ethanol was removed by washing. Recovery was evident 3 h after removal of ethanol (Figure 6A,B). Recovery of mitochondrial function was not dependent on exogenously added NGF and HS (Figure 6C,D).

In addition, we tried to quantify the number of mitochondria in both TMRM and Mito-Tracker stained cells. However, since the fluorescence of these dyes is influenced by mitochondrial membrane potential [29], this quantification may not be accurate, and therefore we did not complete this analysis.

### 2.6. Reversible PS Exposure in Ethanol Treated Neuronal PC12 Cells

**Figure 7:**
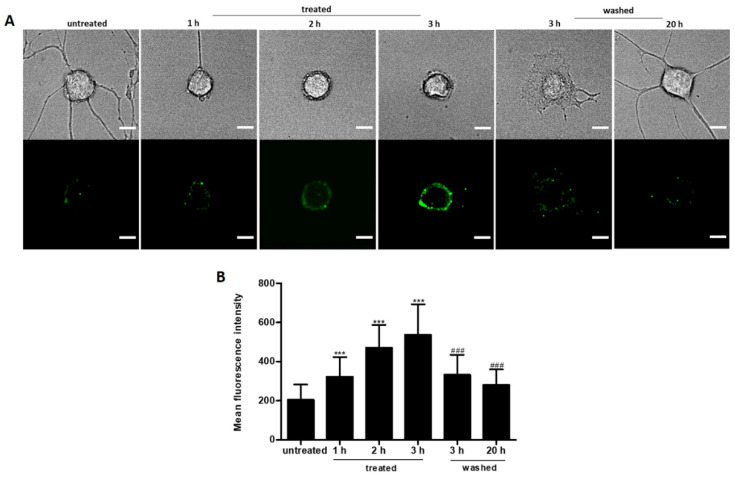
Reversible PS exposure in ethanol treated neuronal PC12 cells. (**A**) live cell imaging of individual cells before ethanol (5%, vol/vol) treatment (untreated), treated with ethanol (treated), and after washing and incubation in fresh medium to allow recovery (washed). PS translocation was visualized by staining with Annexin V-FITC. Scale bar: 20 µm. (**B**) result of quantification of Annexin V-FITC mean fluorescence intensity. Data are presented as the mean ± SD (*n* = 3). *** p < 0.001, compared with untreated group; *^###^ p* < 0.001, compared with the group which was treated for 3 h.

Exposure of phosphatidylserine (PS) on the outer plasma membrane has been well established as an early marker of neuronal apoptosis [30]. Using annexin A5-FITC we observed that neuronal PC12 cells expose PS at the plasma membrane of the cell body after 1 h exposure to 5% ethanol (Figure 7A). PS externalization increased further during 3 h of exposure and was accompanied by neurite retraction. Interestingly, removal of ethanol by washing reversed PS exposure back to base levels within 20 h (Figure 7B). These results demonstrate that PS exposure on the outer leaflet of the plasma membrane is reversible, and that neuronal cells can recover after PS externalization.

### 2.7. Cytochrome C Distribution in Ethanol Treated Neuronal PC12 Cells

**Figure 8:**
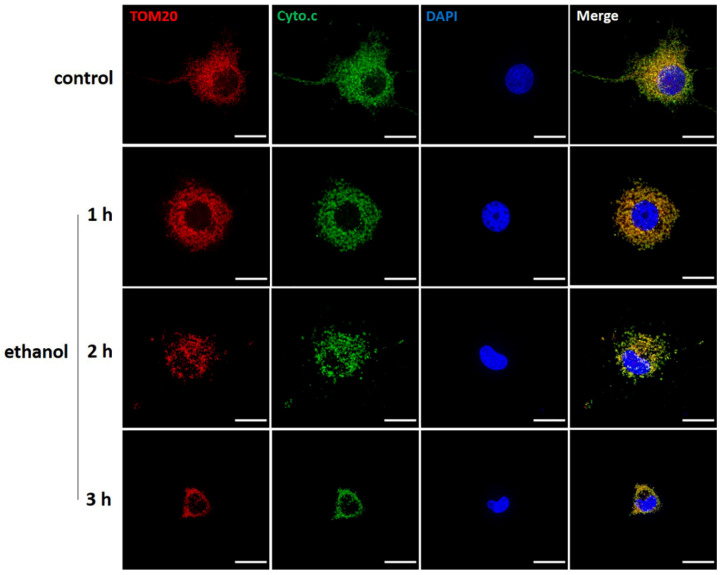
Cytochrome c distribution in ethanol treated neuronal PC12 cells. Confocal microscope images of representative control and ethanol treated (1, 2, 3 h) neuronal PC12 cells, stained with cytochrome c (Cyto.c, green), TOM20 (red) and DAPI (blue). Cytochrome c showed a similar punctuated immunostaining as the mitochondrial staining of TOM20. No indications of diffusion of cytochrome C into the cytoplasm were observed. Scale bar: 50 µm.

Mitochondrial Outer Membrane Permeabilization (MOMP) is a biological process that leads to the release of cytochrome c from mitochondria into the cytosol where it drives caspase activation and cell death [31]. We visualized the distribution of cytochrome c by immunostaining cells with anti-cytochrome c antibody. The morphology of mitochondria was revealed by immunostaining cells with anti-TOM20 antibody. As shown in Figure 8, in untreated cells, cytochrome c was distributed mainly in the mitochondria, which had the normal filamentous shape. After exposure to ethanol for 1, 2, or 3 h, TOM20 immunostaining showed significant fragmented mitochondrial structure. Cytochrome c showed a similar punctuated immunostaining. No indications of diffusion of cytochrome C into the cytoplasm were observed.

## 3. Discussion

In the nervous system, cell death has a large impact because of the limited capacity of mature neurons to regenerate or to be replaced [32]. Here, we report that differentiated PC12 cells exposed to ethanol showed neurite retraction, ROS generation, intracellular Ca^2+^ elevation, nuclear shrinkage and condensation, DNA damage, PS exposure, and mitochondrial fragmentation and dysfunction. These are all major challenges for the neuronal cell and if unresolved, they eventually cause neurons to die [18,19]. Importantly, by monitoring the dynamics of individual cells, we provided evidence that neuronal PC12 cells could survive and recover from all these cell death pathway hallmarks.

The rat pheochromocytoma PC12 cell line is a well-established model system for the study of neurite outgrowth and neuronal differentiation induced by NGF treatment and also is one of the most widely used neuronal cell lines for studying mechanisms associated with neurotoxicity [22]. Upon differentiation, PC12 cells acquire a sympathetic neuron-like phenotype characterized by neurite outgrowth and transition to a post-mitotic state [33,34]. Here we show that ethanol treatment causes significant induction of the cell death pathway in these differentiated PC12 cells in a concentration of 4% or 5% (vol/vol) (Figure 1). Most of the cells died. A short ethanol exposure of 3 h induced about 3% cell death compared to untreated cells (Supplemental Figure S1). After removing ethanol and further culturing in fresh medium for 24 h, no further decrease in cell viability was noticed. Apparently, most cells can survive and recover from a short, 3 h exposure to 5% ethanol.

A clear morphologic sign which occurred within 1 h during ethanol treatment is neurite retraction. Interestingly, when ethanol was removed, neurites quickly regrew (Figure 2). It has been reported that PC12 cell neurite regeneration cannot happen in the absence of exogenous NGF unless there are other interventions like co-culturing with Schwann cells or the overexpression of Nex1 [35,36]. Strikingly, in contrast with these reports, our results showed that the neurite regeneration in ethanol treated neuronal PC12 cells could occur after removing ethanol and was not dependent on NGF or other growth factors provided in the culture medium (Figure 2). Neurite regrowth was also observed when NGF neutralizing antibody was added to the cell culture medium after removing ethanol (data not shown). We cannot completely exclude the possibility of paracrine or autocrine signaling by growth factors produced by the PC12 cells themselves, nor can we exclude that NGF residues remain present at the cell surface after washing. Nevertheless, our results suggest that, once differentiated, PC12 cells do not depend on exogenous NGF for neurite regeneration. In addition, the cell survival after 3 h exposure to 5% ethanol was also not dependent on NGF and HS (Supplemental Figure S1).

ROS and Ca^2+^ are ubiquitous second messengers coordinating a variety of physiological and pathophysiological processes within cells. Biochemical changes and cellular damage that are induced by increased oxidative stress and disruption of Ca^2+^ homeostasis have been linked to the etiology of various neurodegenerative diseases [37]. Raised ROS levels have cellular consequences like lipid peroxidation, protein misfolding and aggregation, DNA damage, and then initiate the cascade leading to programmed cell death [38,39,40,41]. Neurons are also extremely sensitive to Ca^2+^ concentration levels in cellular compartments, and defects in Ca^2+^ homeostasis can alter normal neuronal activity and lead to the activation of cell death pathways [42]. In the present study, we show that there is ROS accumulation and elevation of Ca^2+^ levels in ethanol treated neuronal PC12 cells that accompany the phase of neurite retraction upon ethanol exposure. However, the cells regenerated new neurites and recovered to normal morphology after removing ethanol, which means that cells can recover from this oxidative stress and Ca^2+^ homeostasis disruption (Figure 3). Further study on how neurons can restore normal levels of ROS and Ca^2+^ may reveal attractive targets for the development of neuroprotective drugs.

Mitochondria are the powerhouses of the cell and are involved in various cellular processes, including ATP synthesis, ROS generation, intracellular Ca^2+^ homeostasis, lipid and amino acids synthesis, and regulation of cell death [43]. Changes in mitochondrial structure and function have been linked to various neurodegenerative disorders [44,45]. Mitochondrial dynamics, as well as ultrastructure and volume, are mechanistically linked to mitochondrial function [46]. Abnormal mitochondrial dynamics and fragmentation of the mitochondrial network into small spherical structures are considered hallmarks of mitochondrial injury [47]. We analyzed the mitochondrial network in ethanol treated neuronal PC12 cells and observed that it was highly fragmented, with an increased number of shorter mitochondria and a lower mean mitochondrial length. It is interesting to note that mitochondria fully restored their filamentous structure after removal of the ethanol containing medium and incubation in normal medium, even without exogenous NGF and HS (Figure 5). Previously, reversible neuronal mitochondrial fragmentation was also observed in the neocortex of anesthetized mice after ischemic and traumatic injury revealed by quantitative two-photon imaging [47]. Studies have shown that excessive mitochondrial fragmentation causes ROS generation and cytochrome c release, leading to mitochondrial depolarization and apoptosis [48]. Our results showed not only a massive mitochondrial fragmentation, but also a significant decrease of mitochondrial membrane potential. Importantly, we found that the decreased mitochondrial membrane potential could recover to normal. This recovery was accompanied by increasing mean mitochondrial length and neurite regrowth after the removal of ethanol and was not dependent on exogenous NGF and HS. An explanation could be that injured mitochondria can recover from depolarization or, alternatively, that remaining intact mitochondria multiplied and repopulated in the cell (Figure 6). Further down the road of the cell death program is Mitochondrial Outer Membrane Permeabilization (MOMP). This is a biological process that leads to the release of cytochrome c from mitochondrial intermembrane space into the cytosol, where it drives caspase activation [31]. MOMP is generally considered to be a point of no return because it typically leads to cell death, even in the absence of caspase activation. Our result about cytochrome c immunostaining showed that the distribution pattern of the endogenous cytochrome c in both cell body and remaining neurites appeared to be punctuated and not diffused in the cytoplasm after treating with ethanol for 3 h, which indicated that there was no cytochrome c release from mitochondria at this stage (Figure 8). Previous studies have reported that sympathetic neurons could even survive MOMP [49,50,51]. Because of the crucial role of mitochondria in neuron viability and their important pathophysiological role in neurodegenerative disorders, further dedicated studies are warranted to reveal the mechanisms regulating neuronal survival and recovery following mitochondrial fragmentation, depolarization, and possibly even MOMP.

Externalization of phosphatidylserine (PS) on the surface of the cell membranes of dying cells has been established as an early event on the cell death pathway of neurons [30]. PS is restricted to the inner leaflet of the plasma membrane in normal cells. In our ethanol exposed PC12 cells, PS externalization to the outer leaflet of the cell body membrane occurred, as indicated by labeling with fluorescent annexin 5A. Noticeably, PS exposing neuronal PC12 cells can regenerate neurites and regain normal morphology after the removal of ethanol, suggesting that the loss of membrane phospholipid asymmetry is dynamic and reversible. In addition, this suggests that PS exposing cell bodies remain viable and are able to halt the cell death process and reestablish expansive growth once the inductive stimulus has been removed (Figure 7). Reversible PS exposure has also been observed in degenerated DRG neurons [52], neonatal rat primary cardiomyocytes [53], and myotube formation [54].

Nuclear shrinkage and chromatin condensation are major events along the neuronal cell death pathway and have been recognized as early markers during neuronal degeneration [55,56]. We found not only nuclear shrinkage but also DNA damage in neuronal PC12 cells after treating them with ethanol for only two hours. Importantly, the nuclear morphology recovered to normal accompanied with neurite regeneration after removing ethanol, and DNA damage, as assessed by the comet assay, was also repaired even without exogenous NGF (Figure 4). As post-mitotic cells, neurons have developed an array of DNA repair pathways to resolve specific DNA lesions and maintain genome integrity [57,58]. The repair by these diverse pathways is not always error-free. If DNA damage is not completely repaired, DNA damage will accumulate in the neurons and potentially lead to progressive neurodegeneration, functional loss, or the senescence of neurons [59,60,61,62]. Further research on promoting and improving DNA repair in neurons may help to maintain genome integrity and likely slow down neuronal degeneration.

In summary, our results on PC12 cells differentiated to a neuronal phenotype indicate that the initiation of a neuronal cell death program does not necessarily indicate terminal commitment to final cell death; dying neurons can recover from important hallmarks of the cell death pathway. Removal of the cell death stimulus is sufficient to allow recovery to occur in differentiated PC12 cells, even without adding exogenous NGF, suggesting that this recovery is an intrinsic capacity of neurons. A more in-depth analysis about the precise mechanisms that arrest the neuronal cell death pathway, repair the damage, and restore the normal cellular functions would enhance our understanding of neuronal cell death and provide new potential targets and therapeutic approaches for neurodegenerative diseases.

## 4. Materials and Methods

### 4.1. Cell Culture

Pheochromocytoma line 12 (PC12) cells were obtained from Leibniz Institute DSMZ (ACC 159) and cultured as described before, with minor modifications [22]. Briefly, PC12 cells were maintained undifferentiated in 85% RPMI 1640 medium (61870-010, Gibco, Waltham, MA, USA) supplemented with 10% horse serum (HS; H1270, Sigma-Aldrich, St. Louis, MO, USA), 5% heat-inactivated fetal bovine serum (FBS; F7524, Sigma-Aldrich), and 1% penicillin-streptomycin (pen-strep) antibiotic (Gibco™ 15140122, Thermo Fisher, Waltham, MA, USA) at 37 °C in a 5% CO_2_ incubator. To induce differentiation, cells were dissociated and plated on PDL (50 μg/mL; P6407, sigma, Darmstadt, Germany) and laminin (10 μg/mL; 3400-010-02, R&D Systems, Minneapolis, MN, USA) coated cell culture plate in low serum conditions (1% horse serum, no FBS) and 50 ng/mL of NGF (NGF; N1408, Sigma-Aldrich). Mature neuronal PC12 cells were defined as cells harboring neurites at least two cell body diameters in length [63].

### 4.2. Cell Viability Assay

Cell death was determined by Hoechst 33342 (Sigma) and propidium iodide (PI, Sigma) double fluorescent staining as previously described [64]. PC12 cells were seeded at a density of 1 × 10^5^ cell/mL onto ibidi μ-Slides coated with PDL and laminin, and cultured for six or seven days to facilitate neurite growth. The culture medium was refreshed every two days. After six or seven days, cells were treated with 4% or 5% (vol/vol) ethanol in differentiating medium for 24 h. After treatment, the cells in each group were stained with Hoechst 33342 (5 μg/mL) and PI (5 μg/mL) for 15 min in the dark. The stained cells were observed using Olympus IX81 inverted fluorescence microscopy. Hoechst 33342 stains DNA of living and dead cells and thus labels all cells; PI stains DNA as well, but only that of cells with permeable cell membrane because they are on their path to death. To assess cell viability, 20 visual fields were randomly selected from each group and quantified with Fiji.

### 4.3. Live Cell Imaging and Neurite Analysis

For live cell imaging, we used FEI CorrSight microscopy equipped with wide-field and an Andromeda spinning disk. The FEI MAPS software, in conjunction with Live Acquisition software (LA, FEI) was used to control the microscope and to capture still and time-lapsed images. The microscope was further equipped with an incubation system (Corrsight live module) able to control temperature and CO_2_ levels. Cells were seeded on PDL and laminin coated 6-channel μ-Slides (ibidi, 80606) at a low density of 1 × 10^5^ cells/mL and differentiated for six to eight days; at this low density, individual cells and neurites could be traced. Twenty to 30 fields containing at least 100 cells were chosen randomly for imaging. The same cells were imaged under the microscope before ethanol (5%, vol/vol) treatment (untreated), treated with ethanol for 1, 2, or 3 h (treated), and after washing and incubating in fresh medium with or without NGF, HS and FBS to allow recovery (washed). The ethanol exposure was stopped by discarding the medium containing ethanol, and washing for two times with fresh medium without ethanol. Hereafter, the cells were put back into the incubator for 1 day and the same cells were imaged after relocalization with MAPs. Neurites were defined as processes of more than two cell body diameters in length. Morphological observation of neurite outgrowth was characterized by three different parameters: neurite length, branch number and percentage of cells bearing neurites. Neurite outgrowth parameters were quantified by visual counting and manual tracing with Fiji software. For time-lapse imaging, images were acquired every 10 min; representative images and movies were extracted and edited in Fiji.

### 4.4. Detection of ROS Generation and Intracellular Ca^2+^ Level

For detection of ROS generation, cells were stained with DCFDA (20 µM; ab113851) at 37 °C for 30 min, after which they were washed with fresh culture medium. Images were taken every 5 min with a FEI Corrsight spinning disk; Z-stacks consisted of 10 planes with a Z-interval of 1 µm. For detection of intracellular Ca^2+^ level, cells were incubated with Fluo-8 AM (4 µM; ab142773) for 40 min at 37 °C and washed with fresh medium containing Fluo-8 (2 µM). Images were taken every 30 min before and after exposure to ethanol with a FEI Corrsight spinning disk; Z-stacks consisted of 10 planes with a Z-interval of 1 µm. In addition, bright field images were taken to show morphological changes of the cells. Mean fluorescence intensity was quantified after max intensity projection of z-stacks with Fiji software. Mock-treated cells were imaged in parallel to verify that imaging and staining procedures were not cytotoxic.

### 4.5. Assessment of Nuclear Morphology

Changes of nuclear morphology were detected by staining cells with Hoechst 33342 (Sigma) as described previously [65]. Images were taken at indicated time points with an FEI Corrsight spinning disk; Z-stacks consisted of 10 planes with a Z-interval of 1 µm. Bright field images were taken together to show the cells’ morphological changes. Feret diameter, roundness, and mean fluorescence intensity of Hoechst-stained nuclei was quantified through max intensity projection of Z-stacks with Fiji software.

### 4.6. Mitochondrial Membrane Potential and Mitochondrial Morphology Analysis

Mitochondrial morphology and membrane potential changes were detected by staining cells with tetramethylrhodamine (TMRM, 100 nM; No. 134361, Invitrogen™). Individual cells were monitored under microscopy. Images were taken at indicated time points with a FEI Corrsight spinning disk; the Z-stack consisted of 100 planes with a Z-interval of 0.147 µm. Mitochondrial mean fluorescence intensity was quantified through max intensity projection of Z-stacks with Fiji software. Mitochondrial length was quantified by measuring more than 100 clearly identifiable mitochondria from 20–25 cells with Fiji software.

### 4.7. Detection of Phosphatidylserine (PS) Exposure

Redistribution of PS to the outer leaflet of the plasma membrane was visualized by incubating cells with Annexin V-FITC as described in [66]. Cells were incubated with 1 µg/mL Annexin V-FITC (donated by Prof. Reutelingsperger’s lab; Department of Biochemistry, Maastricht University) in the dark for 15 min at 37 ℃, then washed for fresh culture medium. Images were acquired using a FEI Corrsight spinning disk; Z-stacks consisted of 10 plans with a Z-interval of 1 µm. Bright field images were taken to document the cells’ morphological changes. Mean fluorescence intensity was quantified through max intensity projection of Z-stacks with Fiji software.

### 4.8. Single-Cell Gel Electrophoresis (Comet) Assay

The comet assay was performed under alkaline conditions using a comet assay kit from Abcam (ab238544) following the manufacturer’s instructions. Differentiated PC12 cells were collected with a cell scraper and suspended in cold PBS at a concentration of 2.0 × 10^5^ cells/mL; they were then mixed with low melting agarose at a ratio of 1:10 (*v/v*) and plated onto a comet slide. Alkaline electrophoresis was run at 20 V, 300 mA for 20 min. DNA was stained with Vista Green DNA dye and Fluorescence images were captured using an Olympus BX51 Fluorescence Microscope. Tail length and Olive Tail moment were analyzed for each cell using Comet Assay IV software (Instem).

### 4.9. Immunofluorescence

Differentiated PC12 cells grown on ibidi slides were exposed to ethanol for differentiated time intervals first, then fixed in 4% paraformaldehyde (PFA) for 30 min at 4 °C followed by permeabilization in 0.1% Triton X-100 for 10 min at room temperature, and blocked with 4% Bovine Serum Albumin (BSA) for 1 h at room temperature. For immunostaining, cells were incubated with primary antibodies against cytochrome c (5 μg/mL, Invitrogen, 6H2.B4) and TOM20 (1:50, Cell Signaling Technology, Danvers, MA, USA, D8T4N) overnight at 4 °C. After carefully rinsing in PBS, cells were incubated with a second antibody conjugated with Alexa 488 and 594 (1:500, Invitrogen, Carlsbad, CA, USA) for 1 h at room temperature. The nucleus was labeled by DAPI (Sigma, D9542). The cells were observed and photographed under FEI CorrSight.

### 4.10. Statistical Analysis

Data were obtained from at least three independent experiments and presented as mean ± standard deviation (SD). Values were evaluated by one-way ANOVA followed by a Tukey Kramer test. Prism 5.0 (GraphPad Software, Inc., La Jolla, CA, USA) was used to analyze the data. *p* < 0.05 was considered to indicate a statistically significant difference.

## Data Availability

The data presented in this study are available upon request from the corresponding authors.

## Supplementary Materials

The following supporting information can be downloaded at: https://www.mdpi.com/article/10.3390/ijms23052650/s1.
