# Supplementary material for: Single Cell Analysis of Reversibility of the Cell Death Program in Ethanol-Treated Neuronal PC12 Cells
Source: Int J Mol Sci. 2022 Feb 28;23(5):2650. doi: 10.3390/ijms23052650 (PMC8910107; doi:10.3390/ijms23052650)
Supplement: Supplementary file 1 [file ijms-23-02650-s001.zip › Supplemental Figure S1.pdf]

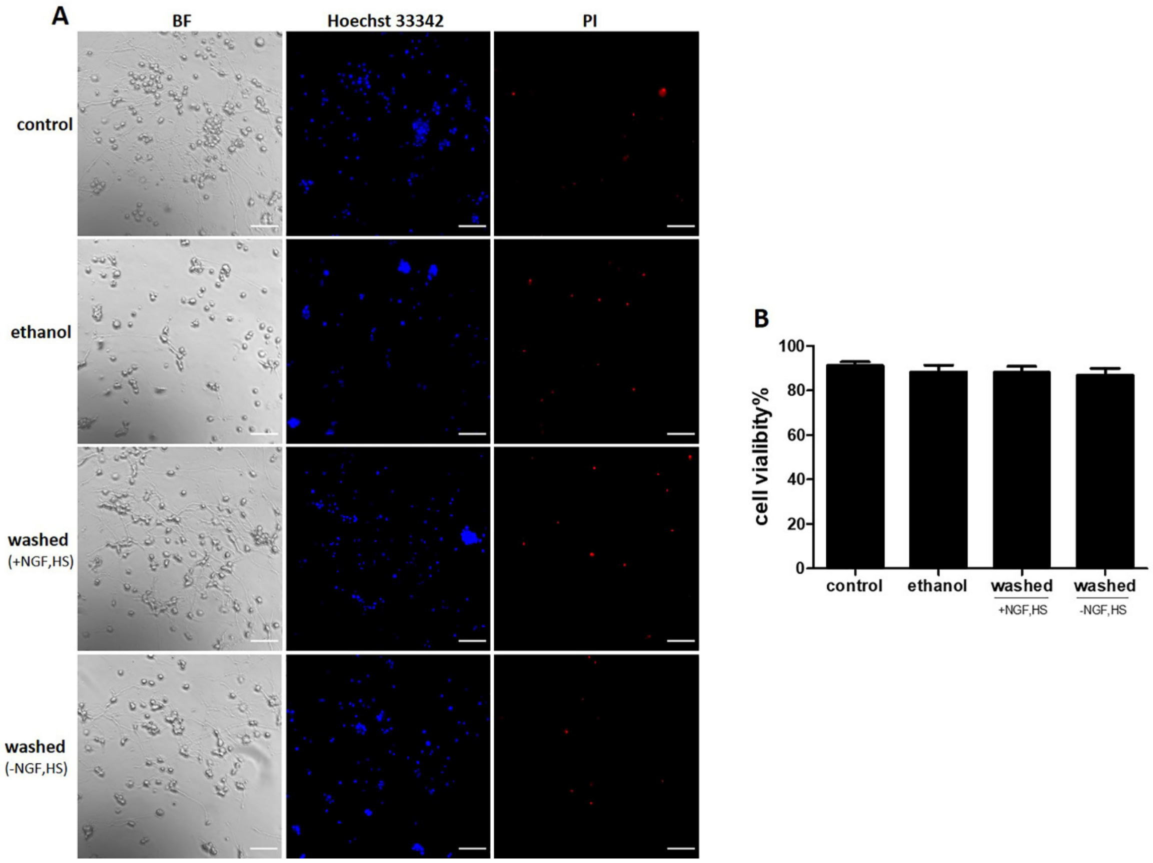

Supplemental Figure S1. Effect of 3 h ethanol exposure on viability of neuronal PC12. Control: untreated cells; ethanol: cells exposed to 5% ethanol (vol/vol) for 3 h; washed: cells exposed to 5% ethanol for 3 h, after which the ethanol was removed by washing with fresh medium and the cell culture was continued in fresh medium either with or without NGF and HS for another 24 h. Cell viability was assessed by Hoechst 33342/PI double staining. Ethanol exposure for 3 h induced about 3% cell death compared to untreated cells. After removing ethanol and further culturing in fresh medium for 24 h, either with or without NGF and HS, no further decrease in cell viability was noticed. Data are presented as the mean ± SD (n = 3). Scale bar: 100 μm.
